# Critical Thinking in English Language Teaching: A Bibliometric Analysis 

**DOI:** 10.12688/f1000research.171160.1

**Published:** 2025-11-25

**Authors:** Edi Sukmojati, Silvia Al Viana, Modjo Kale Djami, Ach Fauzi, Dian Syafirah, Eugenia O.D. Bana

**Affiliations:** 1 English Language Education, State University of Yogyakarta, Yogyakarta, Special Region of Yogyakarta, Indonesia; 2 Master of TESOL, Monash University, Clayton, Victoria, Australia; 3 English Language Education, State University of Yogyakarta, Yogyakarta, Special Region of Yogyakarta, Indonesia; 4 Applied Linguistics, State University of Yogyakarta, Yogyakarta, Special Region of Yogyakarta, Indonesia; 5 English Language Education, State University of Yogyakarta, Yogyakarta, Special Region of Yogyakarta, Indonesia; 6 English Language Education, State University of Yogyakarta, Yogyakarta, Special Region of Yogyakarta, Indonesia

**Keywords:** critical thinking, english language teaching, bibliometrics review, scopus, vosviewer.

## Abstract

**Background:**

Critical thinking (CT) is a fundamental skill in English Language Teaching (ELT) and a key requirement for 21st century education. Despite its pedagogical importance, a comprehensive understanding of research trends and developments in this field remains limited. This study aims to map the evolution of CT research within ELT by examining publication trends, influential authors and journals, thematic patterns, and existing research gaps.

**Methods:**

Bibliometric approach was employed to analyse publications on CT in ELT from 2009 to 2024. Data was retrieved from the Scopus database, and 129 eligible publications were examined using VOSviewer, Biblioshiny (R 4.4.1), and Microsoft Excel. The analysis focused on publication growth, citation performance, author and country productivity, keyword co-occurrence mapping, and thematic structures.

**Results:**

Findings indicate a consistent rise in CT related publications in ELT, with a significant increase between 2009 and 2024. Indonesia was identified as the most productive country, although international collaboration remains limited. The most influential journals include Theory and Practice in Language Studies and the Journal of Language Teaching and Research. Keyword Clustering revealed major themes centred on pedagogy, digital technology, and student engagement, indicating a shift from theoretical discussions toward more applied research directions.

**Conclusion:**

The growing scholarly attention to CT in ELT highlights its increasing academic relevance but also exposes persistent gaps in empirical implementation and cross-national collaboration. Future research should prioritise inclusive, data driven, and longitudinal approaches that integrate pedagogical experimentation and sociocultural perspectives to strengthen CT development in diverse educational settings.

## Introduction

In the current educational landscape, the ability to think critically is widely regarded as a fundamental expectation of English teachers. The enhancement of critical thinking (CT) in higher education institutions represents a complex endeavour, particularly in English as a Foreign Language (EFL) context where cultural, social, and economic diversity shapes classroom interaction ( Gunawardena & Wilson, 2021). Building on this complexity, a sociopolitical perspective has also been integrated into education, particularly in English language teaching, within the framework of Critical Pedagogy ( Curtis et al., 2019).

According to Davies and Barnett (2015), critical thinking involves the cultivation of higher-order cognitive processes such as analysis, evaluation, assessment, synthesis, and comparison. It is widely regarded as a form of reasoning; however, much conventional instruction continues to emphasise argumentation without fostering deeper analytical engagement. Davies and Barnett (2015) further highlight the importance of nurturing students’ disposition toward critical thinking. Accordingly, students must not only acquire cognitive skills but also develop a mindset that encourages inquiry, innovation, and creativity.

The practices of analytical thinking vary across disciplines; in ELT, students are required to analyse the English language critically, reflecting deeply on its structure, use, and application in the construction of meaning ( Pally, 2001). In addition, students must engage with diverse texts, including multimedia formats, and demonstrate the ability to critically analyse and discuss the substance of the subjects they encounter ( Bagattini et al., 2020). However, numerous English Language Teaching (ELT) teachers have not yet developed the reflective competence needed to integrate critical thinking effectively, making the integration of critical thinking into language teaching particularly challenging. Consequently, there is a significant demand for professional development in this area.

Essential competencies in teaching English as a foreign language (EFL) play a crucial role in promoting positive learning outcomes and improving English competence ( Yang & Gamble, 2013). These cognitive abilities empower students to adopt a broader and more reflective perspective on many concerns within the learning process, offering them multiple pathways for exploration and self-directed inquiry ( Marin & Halpern, 2011). Moreover, the usefulness of these skills enables students to identify their own strengths and weaknesses, facilitating self-evaluation of their actions and accomplishments ( Khan et al., 2021). This process of inductive and deductive reasoning, grounded in analysis, synthesis, and evaluation, facilitates the ability to formulate insightful judgments and effectively resolve learning challenges ( Bailin, S., & Siegel, 2003).

To obtain contemporary insights into this growing field, multiple techniques for literature evaluations have been employed to obtain contemporary insights ( Suseelan et al., 2022). Among these, the findings may be methodically presented in a literature review of previous research ( Xiao & Watson, 2019). According to established research questions, researchers can conduct a manual literature analysis utilizing qualitative methods ( Funa & Prudente, 2021), whereas a systematic literature review is typically grounded in several prior investigations ( Angraini et al., 2023). Extending beyond these qualitative approaches, a study by Suseelan et al. (2022) illustrated that, unlike a systematic literature review, a meta-analysis quantitatively synthesizes empirical information from previous research. Specifically, a meta-analysis is a statistical method employed to synthesize the results of multiple studies on an issue, possibly resolving discrepancies among them ( Elyassi Gorji et al., 2021). However, meta-analysis is constrained by its inability to reduce disadvantages through methodology, depending instead on the primary studies’ strategy and availability of correlated data ( Bocconi et al., 2016). As a result, the literature examined in the meta-analysis is relatively consistent ( Aguinis et al., 2011).

More recently, bibliometric analysis is a statistical technique for mapping research trends and evaluating scholarly impact ( Phoong et al., 2022). According to Zyoud et al. (2023), bibliometric approaches help identify the most significant and influential publications in a specific domain. Similarly, Zhang et al. (2019) define bibliometrics as an interdisciplinary technique that integrates science, mathematics, and statistics for the quantitative study of data, generating comprehensive information. This method has become a powerful tool for assessing and clarifying ideas and published information across disciplines ( Rana & Pragati, 2024). Comparable to meta-analysis but broader in purpose, bibliometric analysis enables researchers to synthesise extensive bodies of literature, highlight research gaps, and identify emerging themes ( Suseelan et al., 2022; Chen et al., 2019). In line with this rationale, the present study investigates research on critical thinking within English language instruction from 2009 to 2024.

This study aims to provide an overview of critical thinking research in English language teaching research. This study examines the subsequent research inquiries:

a. To analyse the publication trend concerning “Critical Thinking in ELT,”
b. To investigate the most cited authors of “Critical Thinking in ELT,”
c. To determine the most cited sources related to “Critical Thinking in ELT,”
d. To analyse the most cited countries of “Critical Thinking in ELT,”
e. To investigate the commonly employed keywords associated with “Critical Thinking in ELT,”
f. To propose future research avenues concerning “Critical Thinking in ELT.”

## Methods

### Study design

Bibliometric analysis is an applied research methodology ( Todeschini & Baccini, 2016). Quantitatively, bibliometric analysis evaluates published works, including articles, books, or conferences ( Phoong et al., 2022). Scholars could map the academic field of a particular topic or problem by analysing the quality and quantity of publications, sources, significant contributors, and data linkages, all of which could be done with this technique. Analysis of trends in the field and comprehensive analysis of contributions could be done by utilizing the technological features in Vosviewer, Bibiloshiny, and Microsoft Excel. The methodological framework in this study used descriptive qualitative analysis. Taking relevant bibliometric data sources and collecting the required sources, such as publication titles, years, authors, and institutions, requires significant and accurate identification analysis.

Furthermore, the data was analysed based on related categories or themes, and then the articles’ content included significant findings, methodologies, conceptual frameworks, and patterns that were carefully examined and identified. This research will present more in-depth information and knowledge about the latest publication trends related to the research topic and academic achievements.

### Data collection

**Figure 1.**
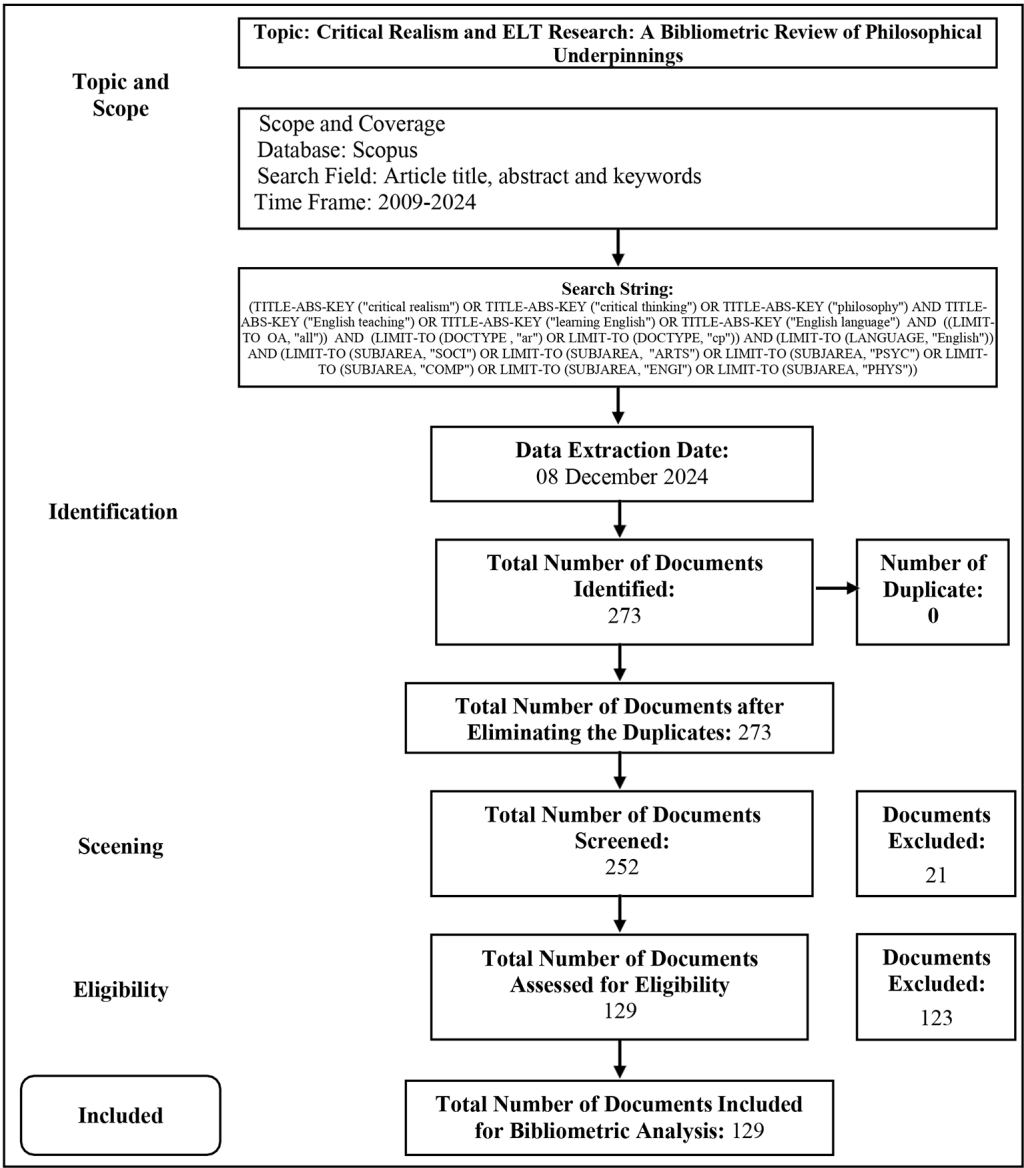
The data collection process.

The researchers utilized the Scopus database to locate data sources of “Critical Thinking in English Language Teaching” because there is extensive interdisciplinary coverage of the field of study- the data acquired was perfected through several stages, as can be seen in Figure 1. The stages include: verification, testing, qualifying assessment, and admission ( Moher et al., 2009).

The first stage was identifying relevant articles using defined search parameters and removing equivalent or duplicated publications. The topic pertained to “Critical Thinking in English Language Teaching.” The investigation into “critical thinking in elt” was restricted to English. 273 publications were detected without duplicates, indicating that only publications with specific criteria can be included as primary data in the extensive collection. The second stage, screening, entails the identification of publications in pertinent languages and document types. English, the most prevalent language for global scientific communication, must fulfil the researcher’s criteria. Only journals and proceedings are permissible for the documentation required for this investigation. Following the screening process, 21 papers were eliminated from the dataset for failing to match the criteria, resulting in 252 publications remaining.

Finally, 252 publications were qualified. The researchers analysed and assessed the title and abstract to find publications that met the inclusion criteria. The main criterion was publications that discuss critical thinking in English language teaching. Publications that meet the requirements could be included in the analysis relevant to the research discourse.

After qualification, 123 publications have been removed for not being relevant to critical thinking in the criteria for English language teaching. In this third phase, 129 publications were left. In this study, the researcher seeks to analyse the landscape and research pattern of critical thinking in ELT, which consists of 129 publications that are guaranteed to be impartial in their interpretive results. These data have been collected during the inclusion phase on December 8, 2024.

### Data analysis

Using a Scopus index database search and following bibliometric analysis, the authors established the publishing trends of critical thinking in ELT. The average number of entire citations was then determined from all the published works with the help of Microsoft Excel technology. In this study, the researcher used the R.4.4.1 program, a bibliometric tool, to find the h-index until the g-index data of publications.

The rankings of journal publications derived from quartiles were significant data displayed using Microsoft Excel software. The data that has been sourced from Scopus’ database includes 129 articles, further categorized into classifications (Q1), (Q2) and (Q3). This data also demonstrated that the publications had been produced by researchers published in journals and conferences.

This conclusion was made by exporting the data to Microsoft Excel, where researchers plot and display the geographical distribution of articles by country on a world map. The g-index and h-index of the publications to be studied were accessed on the publications through the R.4.4.1 program or the use of programs like citation trends on the Biblioshiny. The VOSviewer program created network graphics to define network relationships among the keywords. A survey of key words of critical thinking in English language teaching is carried out with a goal of determining the focus of the research. The information for analysis is collected from the Scopus database and contains preliminarily processed data. The research objective could be understood using the terms offered by the VOS Viewer application.

## Results

### Year-wise publication

This data presents a graph of the ever-increasing research on critical thinking in English Language Teaching (ELT) from 2009 to 2024. From 2009 to 2011, the development is not very high. The growth progressed gradually between 2012 and 2018, though the increase was relatively modest during this period.

**Figure 2.**
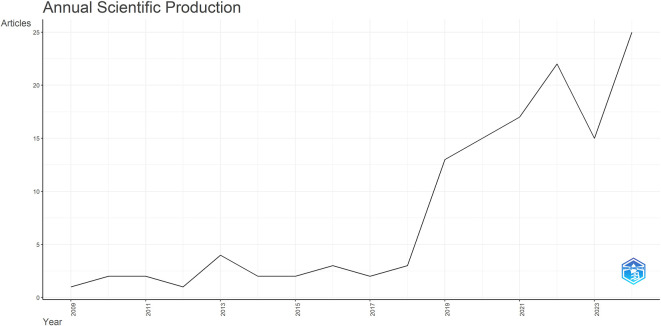
Annual Scientific Production.

A significant increase began in 2019, and the highest was in 2024, reaching 25 articles. This increase reflects the increasing awareness of the role of critical thinking in education. This trend shows a growing focus in ELT, integrating cognitive development through English language learning to meet the demands of increasingly complex and interconnected knowledge. Moreover, this trend also aligns with the pedagogical shift toward 21st-century skills, where Bloom’s Revised Taxonomy emphasizes higher-order thinking analysis, evaluation, and creation as core learning objectives ( Anderson et al., 2001). This pattern is visually represented in Figure 2, which illustrates the annual increase in publications on critical thinking in ELT from 2009 to 2024.

### Authors’ countries publication

This data provides a detailed analysis of contributions to research on critical thinking in English Language Teaching (ELT) from different countries. The analysis evaluates the number of articles published, the percentage represented from the total dataset, and collaborations based on Single Country Publications (SCP) and Multi-Country Publications (MCP). The percentage of MCP indicates articles involving international collaboration.

Indonesia tops the data with 12 articles, accounting for 9.3% of all contributions. The majority are SCPs (11 articles), reflecting a strong focus on domestic research, but only one article involves international collaboration (MCP), accounting for 8.33% of its output. Iran follows with 8 articles (6.2%), of which 2 are MCPs (25%), indicating a greater emphasis on international collaboration than Indonesia. Conversely, China displays 7 articles (5.4%) that are all SCPs, but the data shows no international collaboration. Malaysia and Turkey each show only 6 articles (4.65%). Conversely, Malaysia shows some collaboration, with 16.67% MCPs, while Turkey has no MCPs.

However, four articles from Thailand (3.1%) covered 25% MCP, indicating a similar collaborative pattern to Iran. This was followed by the UK, which contributed three articles (2.3%), all SCP, indicating a lack of international partnership. However, countries such as India, Jordan, Oman, Serbia, South Africa, and the UAE each published two articles (1.55%), all of which were SCP. Several countries, including Belgium, Brunei, Canada, Cyprus, Ethiopia, and Hong Kong, each contributed one article, all of which were SCP, except Ecuador, which stood out with one article that was an entirely international collaboration (100% MCP).

Furthermore, Rana and Pragati (2024) argued that international co-authorship in bibliometric studies correlates with higher citation impact and more sustainable research networks. Such collaborations are also essential for integrating multiple pedagogical and cultural perspectives into the discourse of ELT and critical thinking, which aligns with Gunawardena and Wilson’s (2021) call for socially situated models of critical thinking in multilingual classrooms.

**Figure 3.**
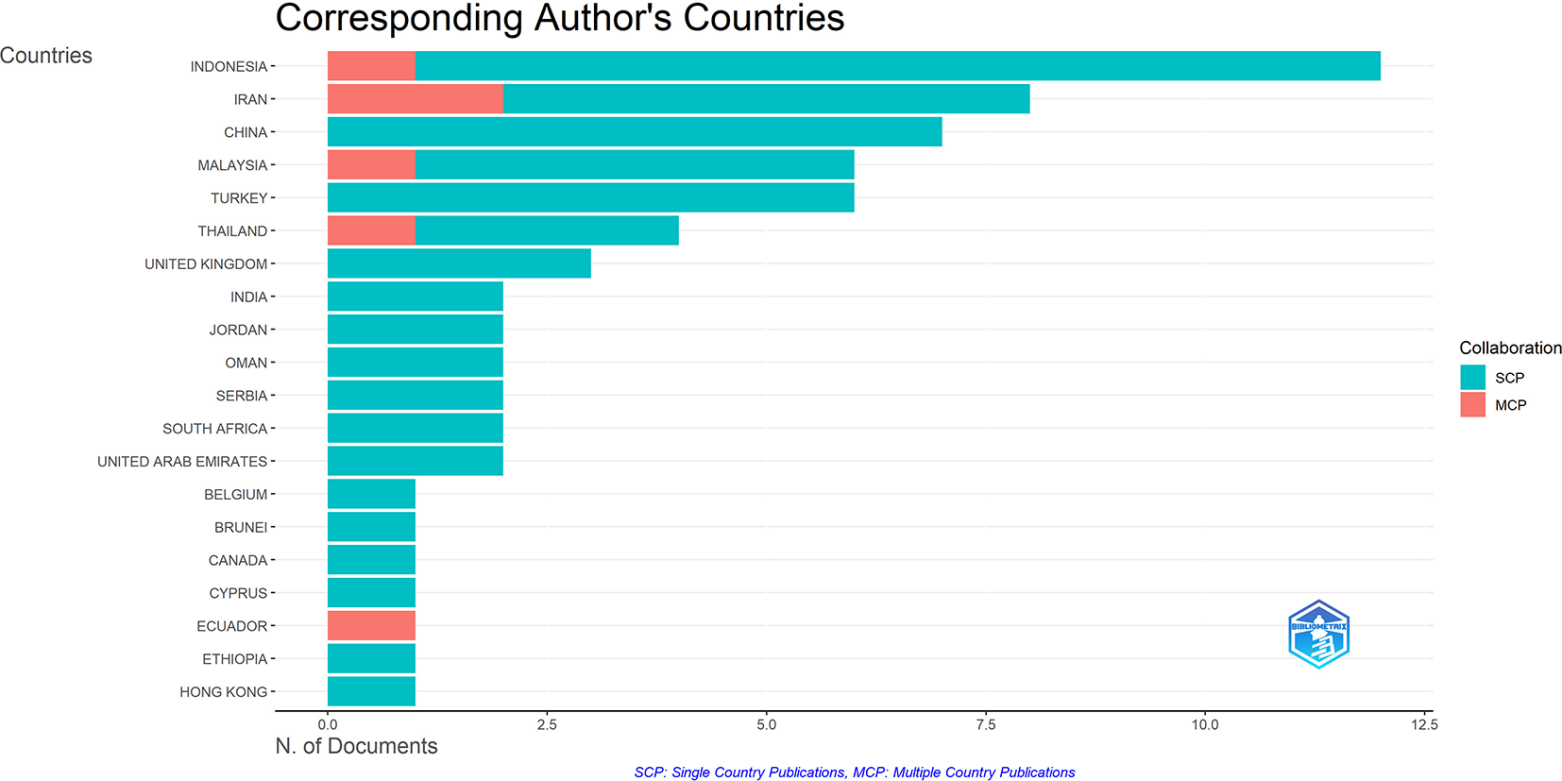
Corresponding author’s countries.

Overall, the data reveals an outlook on research productivity and cooperation at an international level. There are quantities such as Indonesia, which are extremely overwhelming, while there are equals such as the Ecuadoran. Quantities and Qualities of Participation in Global Collaboration Hence, these findings call for extended global cooperation to improve international collaborative research relating to critical thinking in ELT. An overview of these cross-national collaborations and publication outputs is provided in Figure 3, which maps the corresponding authors’ countries and their contribution ratios.

### Journal-wise publications

The findings of these data categories provide an analytical view of the different scholarly journals. The outcome demonstrates how these journals impact and contributes to the thinking area of ELT. The findings captured in this table are h-index, g-index, m-index, total citation (TC), number of papers (NP), and year of the first journal contribution to the field (PY_start). The indicators presented in this data provide insight into these journals’ productivity, impact, and sustainability in the academic landscape. Recent studies by Phoong et al. (2022) emphasize that metrics such as the h-index and g-index are increasingly used to assess not only author productivity but also journal reputation and citation visibility within niche fields like ELT.

In this data, the highest h index was 4, obtained by Theory and Practice in Language Studies, indicating that all four articles have received four or more citations. Then, the highest g-index reached 6 in several journals, such as the Journal of Language Teaching and Research and Theory and Practice in Language Studies. These data show that these journals have frequently cited articles that can increase their visibility. Meanwhile, the m-index showed significant variation; the highest m-index was 0.6, as obtained by the Journal of Language and Linguistics Studies. In addition, BMC Psychology and Computers and Education: Artificial Intelligence scored its highest score of 1, indicating this new journal’s high impact.

However, the number of citations (TC) and the number of papers (NP) provide additional context. Journals such as Procedia - Social and Behavioral Sciences have the highest citations (54) despite their lower output (NP = 3), emphasizing quality over quantity. In contrast, the relatively new World Journal of English Language (PY_start = 2022) has attracted attention for its concentrated efforts in producing impactful works, as evidenced by the m-index of 0.67. Such productivity measures are vital for early-career researchers and curriculum developers to identify credible publication channels, as suggested by Bocconi et al. (2016), who stress the need for aligning research dissemination with journals that support cognitive and pedagogical frameworks in ELT. Detailed bibliometric metrics of the major journals contributing to this field are summarised in Table 1, highlighting h-index, g-index, and citation impact across core publication outlets.

**Table 1.** Sources’ local impact by H index.

Source	h_index	g_index	m_index	TC	NP	PY_start
Theory and Practice in Language Studies	4	6	0.28571429	39	9	2011
International Journal of Applied Linguistics and English Literature	3	4	0.27272727	29	4	2014
International Journal of Language Education	3	4	0.42857143	17	4	2018
International Journal of Media and Information Literacy	3	3	0.375	23	3	2017
Journal Of Language and Linguistic Studies	3	4	0.6	21	4	2020
Journal Of Language Teaching and Research	3	6	0.27272727	36	8	2014
Procedia - Social and Behavioral Sciences	3	3	0.1875	54	3	2009
BMC Psychology	2	2	2	6	2	2024
Cogent Education	2	4	0.4	23	5	2020
International Journal of Instruction	2	2	0.33333333	35	2	2019
Journal of Physics: Conference Series	2	3	0.33333333	26	3	2019
Studies In English Language and Education	2	2	0.4	8	5	2020
World Journal of English Language	2	2	0.66666667	5	4	2022
Advanced Education	1	2	0.25	4	2	2021
Asian-Pacific Journal of Second and Foreign Language Education	1	1	0.5	10	1	2023
Cakrawala Pendidikan	1	1	1	1	1	2024
Cogent Arts and Humanities	1	1	0.2	3	1	2020
Computers and Education: Artificial Intelligence	1	1	1	1	1	2024
Discourse	1	1	0.16666667	27	1	2019
Education and Self-Development	1	1	0.25	3	1	2021

Overall, this data set represents the upcoming nature of critical thinking research in ELT as the journals addressed various platforms from 2009 to the new implication in 2024. These journals show a range of different strategies and levels of prescription, indicating both the spread and development of reflection in ELT research.

### Author-wise publications

In this data, the results of the acquisition are displayed, which include h-index, g-index, m-index, total citations (TC), number of publications (NP), and year of publication (PY_start). The data in this table provide the results of the researchers’ investigation of critical thinking in English Language Teaching (ELT), where all this data was obtained using bibliometric analysis).

The h-index reflects the number of articles (h) published by an author, each of which has been cited at least 1 time. For example, Fahim M and Soozandehfar SMA show h-index results of 2, indicating that they have two articles cited at least twice. Similarly, the g-index measures the distribution of citations among an author’s publications, emphasizing their most cited works. Authors such as Fahim M and Soozandehfar SMA show consistency in their g-index matching the h-index, indicating a limited but focused impact. Moreover, the m-index evaluates the h-index relative to the researcher’s career duration (calculated since PY_start). Authors such as Soozandehfar SMA (0.4) and Alghazo KM (0.5) have higher m-index values, indicating a scientific impact relative to their career duration, while other authors such as Al-Issa ASM (0.09) have lower growth rates of influence over time.

Meanwhile, the number of citations (TC) measures the overall impact of an author’s work. For example, Alkharusi HA has accumulated 18 citations for a single publication, indicating a significant impact despite a low h-index. The number of publications (NP) indicates productivity; authors such as Fahim M and Soozandehfar SMA have two publications, making a small contribution to the field. Meanwhile, some authors have only one publication, indicating lower productivity. Moreover, citation patterns must also be interpreted carefully, as suggested by Bornmann and Daniel (2008), who argued that citation counts are influenced not only by scholarly quality but also by visibility, network effects, and citation culture within disciplines.

However, PY_start indicates the time each author started publishing in the field. Researchers such as Fahim M (2013) and Al-Issa ASM (2014) have been involved in critical thinking in ELT for a long time, while other authors such as AL-Khasawneh F and Alruzzi K (2024) appear to have just started their academic journey. Overall, these data show varying levels of productivity and impact, with some authors emerging as impactful contributors to critical thinking in ELT. This observation reinforces the notion by Glänzel et al. (2003) that bibliometric indicators must be contextualized to properly reflect academic trajectories, particularly in interdisciplinary fields like education. As shown in Table 2, author-specific bibliometric indicators reveal varied levels of productivity and scholarly influence within critical-thinking research in ELT.

**Table 2.** Authors’ local impact by H index.

Author	h_index	g_index	m_index	TC	NP	PY_start
Fahim M	2	2	0.16666667	18	2	2013
Soozandehfar SMA	2	2	0.4	24	2	2020
Abbasian G-R	1	1	0.2	3	2	2020
Abdullah F	1	1	0.25	10	1	2021
Aben Ahmed M	1	1	0.33333333	7	1	2022
Admiraal WF	1	1	0.2	10	1	2020
Aghajani M	1	1	0.16666667	17	1	2019
Al-Alami S	1	1	0.25	1	1	2021
Al-Issa ASM	1	1	0.09090909	13	1	2014
Al-Khasawneh F	1	1	1	1	1	2024
Alghazo KM	1	1	0.5	6	1	2023
Alhaj AA	1	1	0.33333333	1	1	2022
Ali Hih	1	1	0.2	2	1	2020
Alkharusi HA	1	1	0.16666667	18	1	2019
Almansour E	1	1	0.33333333	1	1	2022
Almetov N	1	1	0.5	2	1	2023
Alomery MK	1	1	0.33333333	9	1	2022
Alqaryouti M	1	1	1	1	1	2024
Alruzzi K	1	1	1	1	1	2024

### The occurrence keywords

The visual data presented in this figure shows keywords related to research on critical thinking in English Language Teaching (ELT). This data was obtained using VOSviewer; the network map shows and demonstrates how the terms cluster around the main themes and ideas in the research. Each node represents a keyword, while the relationship between them shows how often these keywords appear together in research publications. The node’s size reflects the frequency of the keyword, and the node’s proximity indicates thematic similarity or strong correlation. The centre of the network is the emergence of the term “ *critical thinking*,” indicating it as a focal point and central theme. This central position shows its important role and high frequency in the discussions as a link to related topics. Several clusters of keywords emerge from this core, forming different thematic clusters that highlight different dimensions of critical thinking in ELT.

Meanwhile, one prominent cluster, marked with a red node, focuses on *students, language learning,* and *perceptions.* Keywords such as “critical thinking skills,” “student perceptions,” and “language learning” indicate an emphasis on how learners engage and develop critical thinking skills in ELT contexts. The cluster also explores connections to methodologies such as “critical reading” and contextual factors such as “COVID-19,” indicating how current global challenges have impacted the field. Moreover, another important cluster, shown in green, covers topics such as *teaching, digital technologies*, and *EFL learners.* This cluster shows a strong connection between integrating critical thinking pedagogy and using technology or digital tools in language teaching. Keywords such as “teaching strategies,” “learning systems,” and “curriculum” indicate an applied focus on teaching design and methodology. Then, the blue cluster highlights terms such as *online learning, active learning,* and *writing skills.* This cluster reflects the growing trend of combining critical thinking with innovative pedagogical approaches, such as distance or hybrid learning environments. This cluster also points to specific skill areas, such as writing, where critical thinking is strongly emphasized.

A recent study by Khoiriyah and Husamah (2018) highlights that students’ perceptions of critical thinking tasks are shaped not only by instructional quality but also by sociocultural classroom environments. Moreover, research by Facione (2020) demonstrates that integrating reflective activities within ELT contexts substantially improves learners’ cognitive engagement, especially in uncertain or disrupted conditions like the pandemic

**Figure 4.**
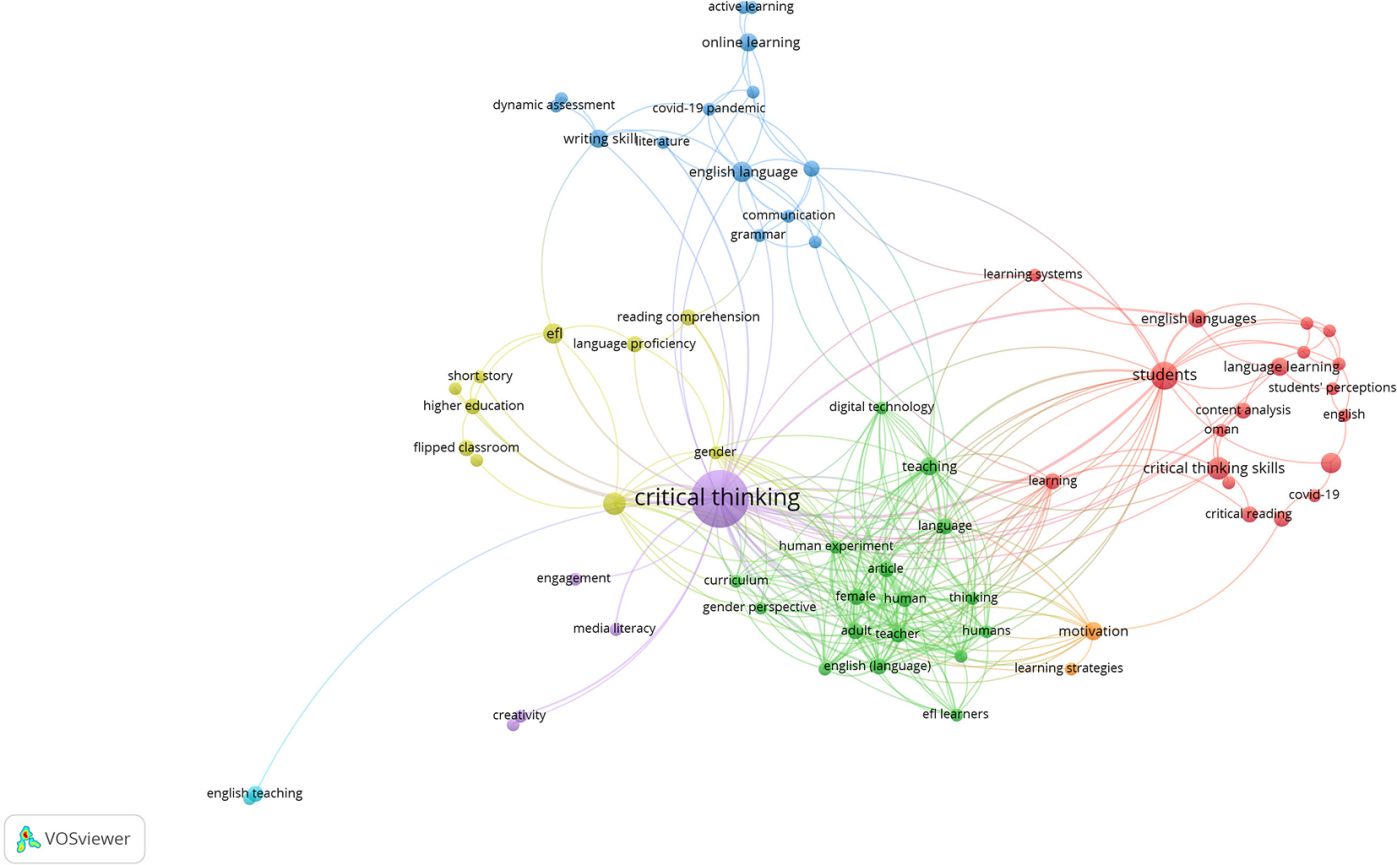
Keywords analysis.

However, the yellow cluster is smaller, focusing on *EFL (English as a Foreign Language), higher education,* and *flipped classrooms.* These keywords reflect the application of strategies in critical thinking to the educational environment, and this keyword also highlights innovations in classroom teaching models so that they impact critical thinking. Finally, the purple and turquoise clusters feature creativity, engagement, and English language teaching terms. These areas show the relationship between critical thinking and broader educational goals, emphasizing how creativity and engagement interplay in developing critical thinking. Furthermore, recent findings by Saavedra and Opfer (2012) confirm that creativity and engagement are integral to critical thinking development, especially when aligned with 21st-century learning competencies. The interconnections among these thematic clusters are visualised in Figure 4, which presents the co-occurrence network of keywords derived from the VOSviewer analysis.

### Thematic map

The thematic map results in this figure show a detailed overview of research themes related to critical thinking in English Language Teaching (ELT), categorized by relevance (centrality) and development (density). Critical thinking emerged as the central and well-developed theme, underlining its important role in ELT research. Its association with terms such as *“adult”* and *“article”* highlights the focus on adult education and the theoretical or practical foundations of the field. The strong position of critical thinking reflects the importance of critical thinking in shaping discourse and methodology in the field.

Meanwhile, themes such as *“students”* and *“teaching”* are also highly relevant but less developed, indicating their fundamental role in implementing critical thinking in the classroom context. These themes highlight the practical integration of critical thinking in pedagogy, particularly through teaching strategies and student engagement. However, their lower density suggests that these areas require further theoretical exploration and empirical studies to strengthen their impact. Additionally, Xiao and Watson (2019) call for more systematic integration of CT into teacher training curricula, emphasizing the need for both declarative and procedural knowledge in pedagogical design.

**Figure 5.**
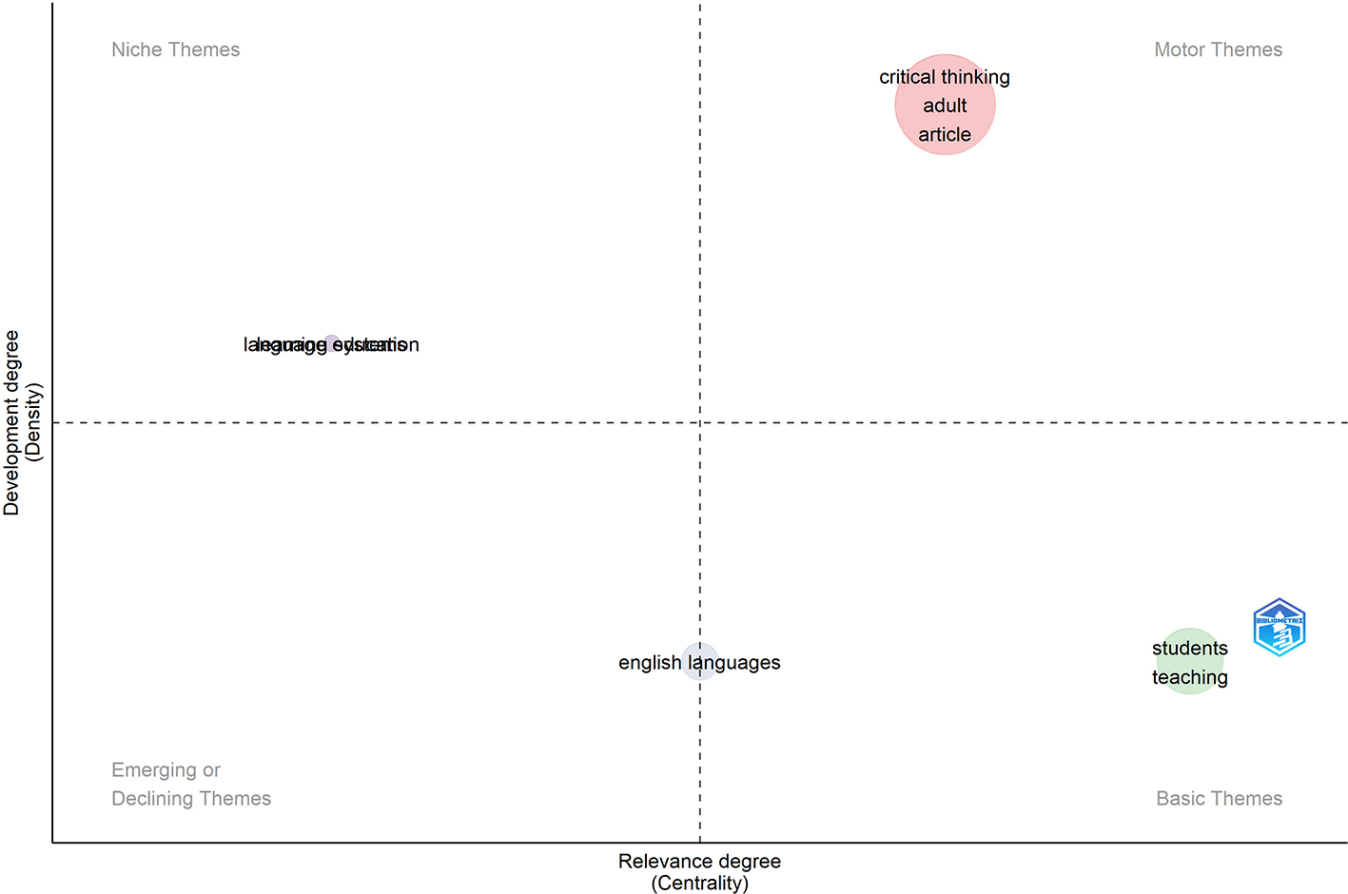
Thematic map.

However, less prominent themes, such as *“English”* and *“language systems,”* show limited integration into critical thinking research or a diminished focus. These topics may stand to gain from the further integration of critical thinking frameworks to boost their importance. In sum, the map locates critical thinking at the core of ELT while reflecting potential weaknesses in the thematic coverage and promising trends for the holistic integration of said theme. The thematic structure and developmental density of these clusters are depicted in Figure 5, providing an overview of the maturity and relevance of research themes in ELT critical-thinking studies.

## Discussion

Critical thinking (CT) in English Language Teaching (ELT) from 2009 to 2024 has garnered significant attention; the research findings indicate uneven development across essential thematic areas. Despite the rising volume of publications, it is apparent that countries, particularly Indonesia and Iran, are concentrating on similar articles instead of participating in international research. This scenario aligns with the assertions of Chen et al. (2019) and Zyoud et al. (2023) that bibliometric concentrations can exist without global links. Despite the expanding body of literature in this study domain, publication distribution remains markedly uneven across countries, necessitating a more equitable and diverse academic discourse ( Aguinis et al., 2011).

The thematic map theoretically indicates a predominant focus on critical thinking, although there is minimal pedagogical integration for students and teachers. This aligns with Defianty and Wilson (2022), who observes that numerous educators lack a comprehensive conceptual framework for critical thinking and predominantly do not possess tools for fostering these constructions in the classroom. The disparity between the examined applications, namely the theory and the translation, exemplifies the research gap. Gunawardena and Wilson (2021) assert that the development of critical thinking is not merely a collection of abilities but a dynamic process that requires assistance, such as recursive instructional design, which remains inadequately addressed in the existing literature. Research by Angraini et al. (2023) further supports this view, demonstrating that integrating critical thinking into grammar and language structure teaching enhances not only linguistic outcomes but also learners’ reasoning abilities.

Moreover, theme-based segmentation is evident in the term cluster: Digital technology and flipped classrooms are emerging; nevertheless, minimal progress has been made in incorporating innovation into evaluation methodologies or reflective teaching methods. This indicates a disparity between educational innovation and cognitive skills assessment systems. Bagattini et al. (2020) and Marin and Halpern (2011) assert that the efficacy of instructional technology should be grounded in cognitive science principles. Consequently, the present study domain may adopt technological trends that overlook essential empirical foundations, potentially leading to significant pedagogical transformations rather than substantial enhancements in learning.

In addition, the data regarding the number of papers in ST on longitudinal and intervention-based studies on CT in ELT indicates a deficiency in this research area, as most of the literature is either descriptive or theoretical. Suseelan et al. (2022) corroborate these findings by demonstrating the absence of longitudinal synthesis in educational bibliometric research. Moreover, while Critical Pedagogy ( Curtis et al., 2019) is frequently characterized as a theory, minimal practical research has been undertaken to implement it through classroom-based investigations. This indicates a deficiency in theoretical application in this context and a lack of critical pedagogy in English Language Teaching, resulting in a shortfall in the transformational potential of critical thinking instruction.

However, there is minimal acknowledgment of the principles of gender intersectionality, socio-economic status, or language variety, which significantly influence critical thinking and education ( Schumer et al., 2018). This study’s limitations lack gender-sensitive or sociocultural analysis, and the deficiencies stem from the insufficient granularity of current bibliometric tools and methodology. Future research should incorporate the development of mixed or more dynamic methodologies concerning the bibliometric technologies employed, including AI-assisted meta-synthesis ( Muthmainnah et al., 2022). Furthermore, these findings underscore the pressing necessity for a pedagogical framework that situates critical thinking as both a cognitive process and a social practice. Socio-Constructivism and Bloom’s Revised Taxonomy are two key ideas that should serve as both reference and guidance in the creation of Critical Thinking in English Language Teaching ( Bailin & Siegel, 2003; Pally, 2001). Research indicates that the implementation of inductive reasoning and dialogical teaching approaches can significantly improve inquiry and reflective assessment in students ( Yang & Gamble, 2013).

This study demonstrates that the CT visibility threshold in ELT research has increased; there are notable deficiencies in collaboration regarding worldwide connections, empirical implementation, and pedagogical consistency. Future research necessitating additional guidance should extend beyond mere descriptive mapping to incorporate theoretical and practical approaches, focusing on classroom-based trials, inclusive frameworks, and cohesive technological pedagogical designs. This aligns with the overarching goals of the ELT, which encompass the cultivation of language proficiency as well as the creation of reflective, socially responsible, and critically engaged learners.

## Conclusion

The study on critical thinking in English Language Teaching (ELT) indicates an important rise in academic publications from 2009 to 2024, indicating an expanding acknowledgement of its significance in education. The bibliometric study provides an international overview of contributions from several countries and authors compared to previous research that predominantly emphasized theoretical frameworks. Although this study focuses on critical thinking in ELT, the domain related to “students” and “teachers” needs further research to show the results of improving its application in the academic environment. This research can reference the encouragement to undertake innovative pedagogical approaches that increase active participation for students, thereby educating students to manage the complexities of the global world and cultivate educated and responsible global citizens. Therefore, further research must continue to investigate relevant approaches in different educational environments.

### Limitations

This research has some limitations; firstly, depending only on the bibliometric data of the Scopus database, it can be argued that the range of publications covered in the literature on critical thinking in ELT is limited. Secondly, the authors tried to eliminate extra irrelevant journals by hand in separate categories of Scopus; however, this step is manual and might contain certain mistakes and non-inclusiveness of certain periodicals. Third, forms of information within the Scopus database exhibit an apparent absence of consistency, particularly regarding author names and institutions. Manual correction was unattainable, potentially affecting our conclusions as the study relies entirely on the quality of the input data obtained from the Scopus database. Fourth, specific evaluations, such as the statistical evaluation of scholars by gender, were impossible in this study due to the technological limitations of the Biblioshiny and VOSviewer tools.

## Ethics and consent

Ethical approval and consent were not required.

## Data Availability

The primary data for this article are the bibliographic references, which are already included in the References section. The extended data in this study are available in the Zenodo repository at [ https://doi.org/10.5281/zenodo.17416524] ( Sukmojati et al., 2025a). Data are available under the terms of the Creative Commons Attribution 4.0 International (CC BY 4.0) license.

## Software availability

The source code used for data processing and analysis was written and executed in R version 4.4.1 [ https://cran.r-project.org/bin/windows/base/] to visualise bibliometric data.

Source code available from: [ https://github.com/achfauzi-web/myresearchcode]

Archived software available from: [ https://doi.org/10.5281/zenodo.17541793] ( Sukmojati et al., 2025b)

License: [ MIT License]

Data visualisations were also generated using VOSviewer [ https://www.vosviewer.com/].
