# Supplementary material for: Critical Thinking in English Language Teaching: A Bibliometric Analysis 
Source: F1000Res. 2025 Nov 25;14:1305. [Version 1] doi: 10.12688/f1000research.171160.1 (PMC12784045; doi:10.12688/f1000research.171160.1)
Supplement: Peer review history

# Reviewer report: Reviewer response for version 1

**DOI:** 10.5256/f1000research.188724.r444775

**Published:** 2026-01-08

**Authors:** Ahmadreza Mohebbi

**Affiliations:** 1 The University of Auckland, Auckland, New Zealand

Major points:

1. Originality and Contribution to the Field:

The article offers a valuable and timely contribution by mapping the evolution of CT research in ELT through a comprehensive bibliometric approach. While bibliometric reviews exist in educational research, few focus specifically on CT within ELT, particularly across such a broad time frame (2009–2024). This fills a noticeable gap in the literature.

1. Methodological Rigor:

The methodology is clearly defined and adheres to standard bibliometric protocols. The authors transparently describe their multi-stage data filtering (Figure 1, p. 5), use of software tools (R 4.4.1, VOSviewer), and criteria for article inclusion. This enhances the reproducibility and validity of the findings.

1. Thematic and Keyword Analysis:

The keyword co-occurrence analysis (Figure 4, p. 10) and thematic mapping (Figure 5, p. 11) are well-executed and provide strong insights into subfields within CT and ELT. Clusters focusing on digital technologies, student engagement, and pedagogical strategies suggest evolving focal points in the literature.

1. Critical Insight into Gaps:

The discussion appropriately highlights areas lacking development, such as limited empirical studies, insufficient cross-national collaboration, and minimal integration of sociocultural or intersectional perspectives. These observations are well-supported by citation data and thematic density maps.

1. Citation and Impact Metrics:

The article offers clear evaluations of influential journals (Table 1, p. 8) and authors (Table 2, p. 9), including h-index, g-index, and m-index scores. This transparency aids in understanding the development and authority within the field.

1. Conclusions and Recommendations:

The conclusions logically follow from the results. The call for longitudinal and intervention-based studies, more diverse theoretical integration, and broader pedagogical experimentation is warranted and constructive.

 Minor points:

1. Terminology Redundancy:

The manuscript occasionally repeats the phrase “critical thinking in ELT” excessively (e.g., in the objectives on p. 4). Consider rephrasing or using pronouns where context is clear to improve readability.

1. Grammatical and Stylistic Edits:

While the manuscript is largely well-written, a few sentences could be tightened for clarity. For instance:

1.
  - “This data presents a graph of the ever-increasing research...” (p. 6) → “The data shows a steady increase in research...”
  - “These keywords reflect the application of strategies in critical thinking to the educational environment...” (p. 10) → Consider simplifying or splitting this sentence for clarity.
2. Limited Qualitative Insight:

While bibliometric studies are quantitative by nature, the manuscript could benefit from qualitative commentary on *how* CT is being conceptualized across major themes or regional contexts. This might offer richer pedagogical insights.

1. Software Versions and Citations:

The authors correctly cite the use of VOSviewer and Biblioshiny, but they could briefly justify why these tools were chosen over alternatives (e.g., CitNetExplorer, Gephi) to emphasize methodological transparency.

1. Scope of Literature Sources:

The study focuses exclusively on Scopus-indexed literature. While this is common in bibliometrics, the authors rightly note this as a limitation. They might briefly mention how this could affect the representation of non-Western or emerging research.

 Suggested Experiments or Future Work:

- Empirical Pedagogical Interventions:

Encourage future researchers to move beyond bibliometrics into classroom-based research testing CT-enhanced ELT strategies, particularly in underrepresented regions (e.g., Sub-Saharan Africa, South America).

- Mixed Methods Meta-Synthesis:

Combine bibliometric findings with qualitative content analysis of selected high-impact studies to reveal the depth of pedagogical theory and CT implementation.

- Longitudinal Tracking:

Propose tracking CT skill development over time in ELT classrooms using standardized critical thinking rubrics (e.g., from the Delphi Report or Facione’s framework).

- Cross-national Collaboration Studies:

Develop co-authored studies between high-output but isolated countries (e.g., Indonesia, Iran) and nations with strong ELT traditions but fewer CT-ELT publications.

Is the work clearly and accurately presented and does it cite the current literature?

Yes

If applicable, is the statistical analysis and its interpretation appropriate?

Yes

Are all the source data underlying the results available to ensure full reproducibility?

Yes

Is the study design appropriate and is the work technically sound?

Yes

Are the conclusions drawn adequately supported by the results?

Yes

Are sufficient details of methods and analysis provided to allow replication by others?

Yes

Reviewer Expertise:

Cultural Linguistics, AI in education, Mixed methods research

I confirm that I have read this submission and believe that I have an appropriate level of expertise to confirm that it is of an acceptable scientific standard.

# Reviewer report: Reviewer response for version 1

**DOI:** 10.5256/f1000research.188724.r444774

**Published:** 2026-01-02

**Authors:** Abdul Khalique Khoso

**Affiliations:** 1 Yangzhou University, Yangzhou, Jiangsu, China

The manuscript provides a timely and relevant bibliometric analysis of a growing field. The research design is appropriate, and the data visualization is clear. However, the manuscript suffers from significant issues in clarity, structure, and depth of analysis. The writing is often verbose and repetitive, key concepts are not well-defined, and the discussion fails to synthesize the results into a coherent, impactful argument. Major revisions are required to enhance its rigor and scholarly contribution. My comments are given below which will help authors to bring clarity and consistency in it. 

 1. The introduction is lengthy but does not clearly articulate the unique contribution of this bibliometric analysis. It extensively reviews methods of literature review (systematic review, meta-analysis) but does not convincingly state why a bibliometric analysis is the necessary next step for this specific topic. The stated aim "to provide an overview" is too vague. What does this overview reveal that wasn't known, and why does it matter?

 2. Sharply define the research gap: What is missing from our bibliometric understanding of CT in ELT? Cite existing bibliometric reviews in related fields to position your work.

 3. Shorten the methodological comparison. Focus on why bibliometrics is the right tool to answer your specific research questions. End intro with a clear, concise statement of contribution.

 Methodology

 4. The search string "Critical Thinking in English Language Teaching" is overly narrow and likely missed many relevant publications that use synonyms (e.g., "critical thinking in EFL/ESL," "higher-order thinking skills in ELT"). This casts doubt on the comprehensiveness of the dataset (n=129).

 5. The manuscript states the topic was "Critical Thinking in English Language Teaching" but does not provide the exact search string used in Scopus (e.g., TITLE-ABS-KEY("critical thinking" AND "english language teaching")). This must be explicitly stated for replicability. Suggestion: Provide the exact Boolean search string used in the Scopus database in the "Data collection" subsection. 

 6. Clearly explain the roles of VOSviewer (network analysis), Biblioshiny (performance analysis), and Excel (descriptive stats). The use of VOSviewer, Biblioshiny, and Excel is listed but not justified. The rationale for using multiple tools and what specific analysis each tool was used for is unclear. Briefly justify the choice of each software (e.g., VOSviewer for network visualization, Biblioshiny for performance analysis) and map which tool was used for each reported result (Figures 2-5, Tables 1-2).

 7. The screening process (Figure 1) mentions removing 21 documents for language/type, then 123 for relevance. The criteria for "relevance" are vague. my suggestion is to Specify the inclusion/exclusion criteria applied during the title/abstract screening (e.g., empirical studies, theoretical reviews, specific ELT contexts) to clarify how relevance was determined.

 8. The analysis description omits standard bibliometric steps, such as data cleaning (e.g., author name disambiguation) and normalization of citation counts. my suggestion is, describe the data cleaning steps undertaken. Acknowledge the limitation of author name inconsistencies mentioned later in the limitations as a known constraint of the method.

 9. The discussion often restates findings (e.g., "Indonesia was the most productive country") without synthesizing them to answer the "why" or "so what." here move beyond description. For example, discuss why Indonesia might be the most productive (e.g., national education policy, research funding focus) and the implications of limited international collaboration for the field's development.

 10. The discussion does not systematically address each of the six research questions posed in the introduction. The answers are scattered and implicit. Structure the discussion with sub-sections that directly correspond to and answer each research question (e.g., 5.1 Publication Trends, 5.2 Influential Authors and Sources).

 11. The discussion is very good in terms of comparing findings with previous literature for which authors are highly appreciated. here i would suggest few more studies which should be cited in discussion which will support your claims more strongly, study1:  Khoso, A.K., Honggang, W., Darazi, M.A. Trust and attitude towards AI as pathways to creativity: a TAM Model study of EFL students’ digital literacy and AI acceptance. Humanit Soc Sci Commun (2025). https://doi.org/10.1057/s41599-025-06362-x.   study 2: Khoso, A. K., Honggang, W., & Darazi, M. A. (2025). Empowering creativity and engagement: The impact of generative artificial intelligence usage on Chines EFL students' language learning experience. Computers in Human Behavior Reports, 18, 100627. https://doi.org/10.1016/j.chbr.2025.100627. 

 I appreciate the authors’ efforts in addressing an important topic. I hope that my comments and suggestions will help strengthen the manuscript and guide its potential revision.

Is the work clearly and accurately presented and does it cite the current literature?

Yes

If applicable, is the statistical analysis and its interpretation appropriate?

Yes

Are all the source data underlying the results available to ensure full reproducibility?

Yes

Is the study design appropriate and is the work technically sound?

Yes

Are the conclusions drawn adequately supported by the results?

Yes

Are sufficient details of methods and analysis provided to allow replication by others?

Yes

Reviewer Expertise:

English Language Teaching (ELT) AI and Education

I confirm that I have read this submission and believe that I have an appropriate level of expertise to confirm that it is of an acceptable scientific standard, however I have significant reservations, as outlined above.

# Reviewer report: Reviewer response for version 1

**DOI:** 10.5256/f1000research.188724.r438900

**Published:** 2025-12-31

**Authors:** Siti Drivoka Sulistyaningrum

**Affiliations:** 1 Universitas Negeri Jakarta, Jakarta, Indonesia

The manuscript establishes a robust bibliometric framework utilizing standard tools such as VOSviewer and Biblioshiny. The methodology is transparent, and the findings concerning the increase in publications and the productivity of Indonesian authors are evident. Nevertheless, the quality of writing can be greatly improved by transitioning from "descriptive reporting" (articulating the data) to "analytical synthesis" (interpreting the implications of the data). The academic register requires refinement to enhance its authority.

**Abstract**

 "Bibliometric approach was employed to analyse publications..." Sugested revision

 "A bibliometric approach analysed publications..." (Active voice is stronger).

**Results**

 "This data provides a detailed analysis of contributions..." sugested revision

 "An analysis of country-specific contributions reveals distinct productivity patterns."

 Enhancing Cohesion and Flow paragraph transitions frequently sound abrupt. For pointing out the reader, use "signposting" terminology. Connecting Findings: Use a bridge sentence to go from "Countries" to "Journals."

 Idea: "Having established the geographical dominance of Asian contexts, it is necessary to examine the specific publication outlets facilitating this discourse. "Explaining "Critical Pedagogy" The gap Critical pedagogy is mentioned in the introduction, and a dearth of useful research on it is noted in the debate.

 Take action: strengthen this thread. Make it clear in the Results section (Keyword Analysis) that although "critical thinking skills" are prevalent, there are no keywords pertaining to "political" or "social justice" parts of critical pedagogy. This is a fantastic way to set up your conclusion.

Is the work clearly and accurately presented and does it cite the current literature?

Partly

If applicable, is the statistical analysis and its interpretation appropriate?

Partly

Are all the source data underlying the results available to ensure full reproducibility?

Partly

Is the study design appropriate and is the work technically sound?

Yes

Are the conclusions drawn adequately supported by the results?

Partly

Are sufficient details of methods and analysis provided to allow replication by others?

Partly

Reviewer Expertise:

ICT in ELT, Pedagogical Skills, Integrating Curriculum Material Development with 21st century skills.

I confirm that I have read this submission and believe that I have an appropriate level of expertise to confirm that it is of an acceptable scientific standard, however I have significant reservations, as outlined above.
